# Malignant Otitis Externa in a 20-Year-Old Male Patient With Erdheim-Chester Disease: A Case Report

**DOI:** 10.7759/cureus.20498

**Published:** 2021-12-18

**Authors:** Yasser Ghazi S Alarimah, Khalid Athal H Alanazi, Nouf H Alshammari, Hanadi A Fatani, Nader F Aldajani

**Affiliations:** 1 Otolaryngology-Head and Neck Surgery, College of Medicine, Almaarefa University, Riyadh, SAU; 2 Otolaryngology-Head and Neck Surgery, King Fahad Medical City, Riyadh, SAU; 3 Head and Neck Pathology, King Fahad Medical City, Riyadh, SAU

**Keywords:** otolaryngology-head and neck surgery, erdheim-chester disease, non-langerhans histiocytic, neuro-otology, multisystem disease, malignant otitis externa

## Abstract

Malignant otitis externa (MOE) is an uncommon, potentially life-threatening, invasive infection of the external auditory canal and lateral skull base. It is mainly seen in older adults with diabetes or immunocompromised patients and rarely seen in children.

Erdheim-Chester disease (ECD) is a rare, non-Langerhans histiocytosis disorder. It is a multisystem disease with a poorly understood etiology. It commonly affects the skeletal system, central nervous system (CNS), cardiovascular system, lungs, retroperitoneum, kidneys, and skin. CNS involvement confers poor prognosis and reduced response to treatment. Cardiovascular involvement is another indicator of poor prognosis.

This report describes the case of a 20-year-old male with ECD who had bilateral malignant otitis externa/skull base osteomyelitis and hearing loss. The patient was not responding well to treatment. He was on intravenous antibiotics, underwent left mastoidectomy, received targeted immunotherapy, and had four rounds of chemotherapy. The patient died after six months from the diagnosis and targeted treatment, which indicates the aggressiveness of the disease.

MOE should be suspected in any patient presenting with intractable otalgia with otorrhea that is not responsive to local treatment for uncomplicated otitis externa or otalgia with temporomandibular joint (TMJ) pain aggravated by chewing.

This is the first report of malignant otitis externa in a young patient with ECD to the best of the author's knowledge. This case emphasizes the importance of suspecting MOE in young patients, especially if the risk factors of the disease are present because early diagnosis can prevent or minimize life-threatening complications.

## Introduction

Erdheim-Chester disease (ECD) is a rare, non-Langerhans histiocytosis disorder. It was first described in 1930 by Dr. William Chester and Dr. Jakob Erdheim, and there have been around 600 cases reported in the literature since then. ECD cases have increased dramatically in the last decade due to increased physicians' awareness about the diagnosis and, recently, It has been reclassified as histiocytic dendritic cell neoplasm [1]. It is a multisystem disease with a poorly understood etiology; most articles in the literature reported proto-oncogene *BRAF* V600E mutation in patients with ECD between 38% and 68%, and 100% using more sensitive tests in a recent article, which strongly supports possible clonal origin [2]. It has been observed that ECD histiocytes express a pattern of proinflammatory cytokines and chemokines, characteristically found in the sera of ECD patients. This pattern shows elevated levels of Interferon-alpha (IFN)-a, Interleukin (IL)-12, and monocyte chemotactic protein-one. It also shows a decrease in the levels of IL-4 and IL-7 [2].

The disease is associated with significant morbidity and typically affects adults between the fifth and seventh decades with a slight predominance in males [3]. It commonly affects the skeletal system, central nervous system (CNS), cardiovascular system, lungs, retroperitoneum, kidneys, and skin. The skeleton is involved in 96% of ECD patients, with osteosclerosis being the classical hallmark of skeletal involvement, but only 50% of the patient complain of bone pain [3]. CNS involvement confers poor prognosis and reduced response to treatment. Symptoms occur in up to 50% of patients, commonly causing eye disorders, cerebellar syndrome, diabetes insipidus, seizures, and radiculopathy [4]. Like CNS involvement, and depending on the size and location of the lesion, cardiovascular involvement is another indicator of poor prognosis; frequently seen lesions include pericarditis, perivascular infiltration, and periaortic fibrosis. Other common conditions such as retroperitoneal fibrosis, pulmonary infiltrates, and xanthelasma, can occur; however, there is significant variation in organ involvement and severity of illness, resulting in numerous clinical presentations and symptomatology. To this end, patients can initially present with exclusively CNS symptoms and subsequent neuroimaging may demonstrate homogeneously enhancing and discrete tumor-like lesions, such as meningioma and cranial nerve schwannomas [3].

The rarity of ECD and the lack of a classical picture make the diagnosis extremely challenging. The average time to reach the diagnosis is between a few months to several years. ECD diagnosis is based on thorough clinical evaluation, a detailed patient history, identification of characteristic symptoms, laboratory studies, and many specialized tests. Such studies may include plain x-rays as well as advanced imaging techniques, including computed tomography (CT), magnetic resonance imaging (MRI), 99mTc bone scintigraphy, and 18F-fluorodeoxyglucose (FDG)-positron emission tomography (PET). Although FDG-PET/CT is less sensitive for bone lesions, it remains the preferred imaging modality for the diagnosis due to its ability to assess organ involvement. Plain x-rays of involved bones typically reveal symmetrical increased hardening and thickening mainly in the metaphysis and diaphysis, with sparing of the epiphyses. This finding is considered distinctive of ECD.

A tissue biopsy is highly recommended even when the clinical and imaging features are suggestive of ECD, not only for diagnosis but also for identifying any associated mutations. Molecular testing of the biopsy may reveal mutation or alteration in the genes of PI3K-AKT or MAPK-ERK pathway, such as *BRAF* mutation, which can change the focus of treatment plans. Histopathology examination of tissue samples usually shows infiltration by foamy histiocytes, fatty (lipid)-laden with certain non-Langerhans cellular features, and large, distinctive cells with multiple nuclei (Touton giant cells) [5].

According to the currently available literature, the skull base is rarely involved in ECD. In documented cases of ECD of the skull base, the central and the lateral skull base were the most frequently affected [6]. Although it can be involved at any age, patients are typically middle-aged with a substantial male predominance. Common clinical presentations include diplopia, dysarthria, headache, vertigo, hearing loss, facial nerve paresis, trigeminal neuralgia, and trigeminal hypesthesia. ECD of the skull base may also be associated with systemic symptoms, tumor-like lesions that mimic other skull base tumors, and CNS findings on neuroimaging. The diagnosis of skull base ECD is complex, and it is almost indistinguishable from other skull base lesions by neuroimaging alone. However, homogenous enhancement of the mass on T1-weighted post gadolinium MRI was found to be the most consistent finding in ECD of the skull base. T2 hypointensity may also be observed in some patients [6]. The treatment of skull base ECD should be tailored for each patient; some patients may need corticosteroids, INF-a, or vemurafenib in *BRAF* v600E mutations. It is also important to mention that surgical resection is not curative, and it carries a significant risk of neurovascular injury [6].

After prolonged searching in the literature, we found no mention of any associating between ECD and malignant (necrotizing) otitis externa (MOE). This is the first report of malignant otitis externa associated with ECD to the best of our knowledge. MOE is an uncommon, potentially life-threatening, and invasive infection of the external auditory canal and lateral skull base, which can progress to the adjacent structures through the osseous-cartilaginous junction (Fissures of Santorini). Osteomyelitis of the skull base and temporomandibular joint (TMJ), or cranial nerve palsies, are signs of progression and an advanced infection [7]. MOE should be suspected in any patient presenting with intractable otalgia with otorrhea that is not responsive to local treatment for uncomplicated otitis externa or otalgia with TMJ pain aggravated by chewing. It is mostly seen in older adults with diabetes or immunocompromised patients, and it is rarely seen in children.

*Pseudomonas aeruginosa* is responsible for almost all MOE cases (>98%) and fungi or mucus-cutaneous microbiota in the rest [7]. Malignant otitis externa is associated with many serious complications, increased morbidity and mortality rates, intracranial invasion, and cranial nerve involvement, most commonly affecting the facial nerve (VII), followed in order by glossopharyngeal (IX), vagus (X), accessory (XI), and hypoglossal nerve (XII) rarely [8].

## Case presentation

Our patient is a 20-year-old male who was not known to have any chronic medical illness before his initial presentation to ER. He complained of severe left ear pain with discharge for a few days, and it was treated medically as an acute left otitis externa. A few days later, the patient was admitted to his local hospital because he was not improving. He was started on IV antibiotics in the first few days of the admission. The investigation proceeded for radiological and nuclear studies (CT, MRI, bone scan, gallium scan), which confirmed osteomyelitis of the left temporal bone and skull base and active otomastoiditis. He was started on antipseudomonal antibiotics (ciprofloxacin) with infectious disease consultation and follow-up. The patient continued his admission without satisfying clinical and radiological improvement until more than three months and was discharged on oral ciproflocaxillin with Out Patient Department (OPD) regular follow-up. During follow-up, the left ear complaint was only ear pain, which improved after the treatment course, and the antibiotics duration was given for a total of 18 weeks.

After eight months, he presented to OPD complaining of severe right ear pain and ear discharge. In addition, there was complete hearing loss in the left ear, which was not present before. Upon evaluation, it was found that he had the same signs and symptoms as in his previous admission, including bilateral edematous mucosa, minimum discharge, and pain. This time the symptoms were in the right ear with signs of otitis externa and otomastoiditis. He was admitted and started on antipseudomonal antibiotics (ciprofloxacin) with analgesia, and was referred later to the otologist for mastoid core biopsies. Left simple mastoidectomy was done, as the disease is still radiologically present in both sides, and it was the worst hearing ear.

**Figure 1:**
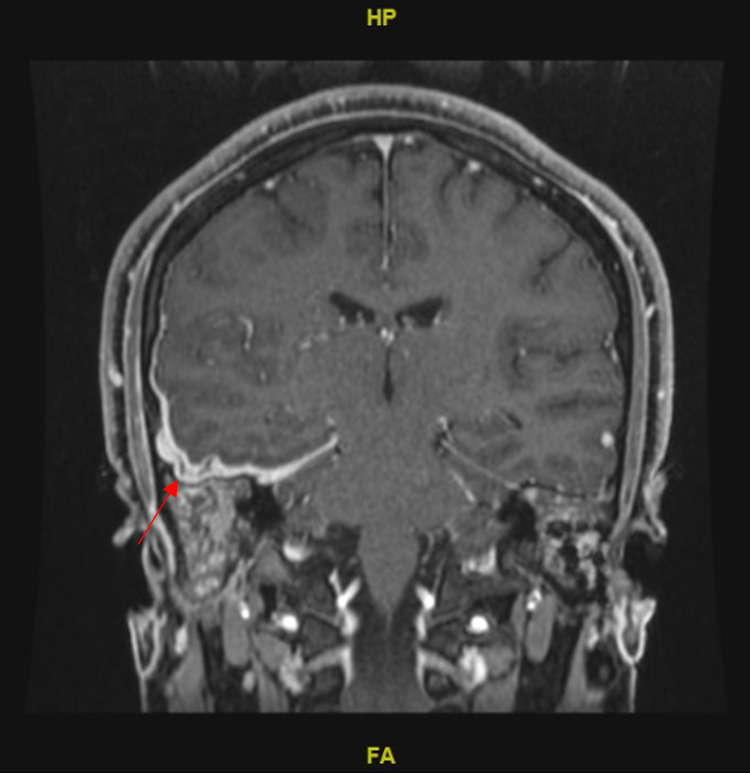
Thickening of the pachymeninges (red arrow)

There was no isolated organism from his left or right ear, and biopsies taken from left mastoidectomy were negative for bacterial, fungal, and TB. Histopathology showed only inflammatory cells. During admission, the patient started to complain of headaches with photosensitivity. Fundoscopy examination showed optic disc edema, and brain MRI was requested and showed signs of meningitis, confirmed by CSF analysis. Head MRI showed bilateral petrous and mastoid destructive changes and mastoiditis, with membranous labyrinthine enhancement. Those changes were observed more on the right side, with pituitary stalk, leptomeninges and pachymeninges thickening (Figure 1), and bilateral intraocular optic disc protrusion. 

During the hospital stay, he complained of progressively increased water intake for two weeks associated with headaches. He was seen by the nephrologist and the endocrinologist, diagnosed with diabetes insipidus, and started desmopressin. He also developed facial nerve palsy, for which he was started on steroids. Interestingly, most of his symptoms resolved after receiving the steroid, including headache, ear pain, and facial palsy.

Head and neck CT was requested and showed soft tissue thickening, subcutaneous air foci, and fluid effusion over the left mastoidectomy site, opacity in the middle ear cavity, and external auditory canal. Also, their opacity due to fluid effusion was noted in the right middle ear and mastoid air cells. The neck structures were unremarkable, and there were no masses or lymphadenopathy. Also, there was no intrathoracic, abdominal, or pelvic involvement. 

The patient was then referred to our tertiary hospital in King Fahad Medical City, Riyadh, where he was admitted for further investigation and management. He was complaining of bilateral leg pain upon his arrival, and his symptoms became worse after finishing the course of oral steroids. We suspected was an immunological disease, and consulted a rheumatologist for it. 

Multiple immunological investigations were done, including: dsDNA, C3, which were within normal range, C4 was slightly elevated (0.409) (g/L). Anti-neutrophil cytoplasmic antibodies (ANCA) myeloperoxidase (MPO) level was normal, and ANCA proteinase 3 (PR3) was slightly elevated (26.6) EU/mL

**Figure 2:**
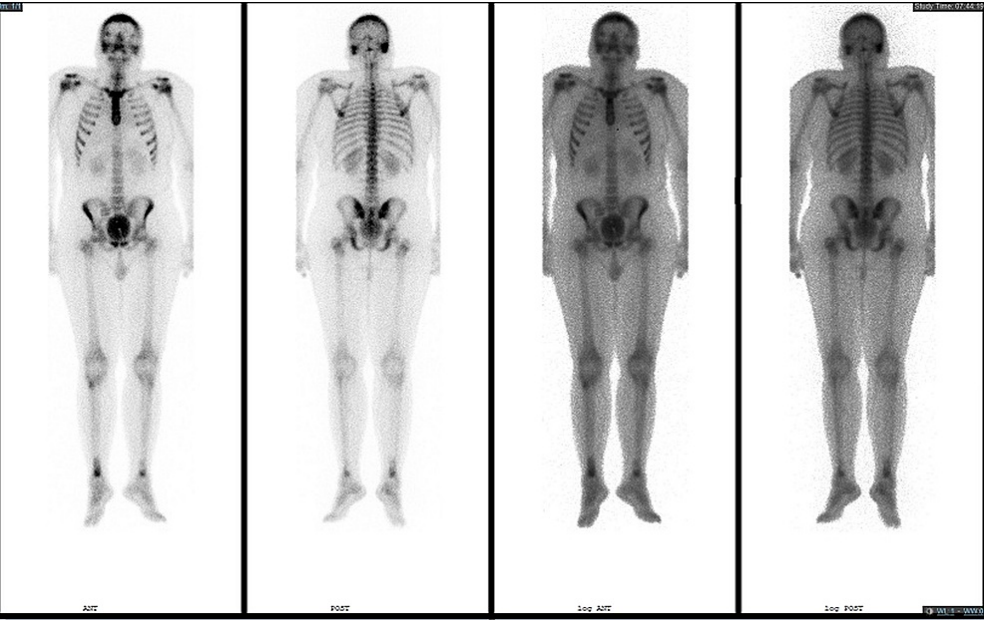
Tc-99m-MDP bone scintigraphy showed bilaterally increased uptake within the distal tibia, more prominent on the right side. Plus, the high uptake in both lateral skull bases Tc: technetium; MDP: methylene diphosphonate

**Figure 3:**
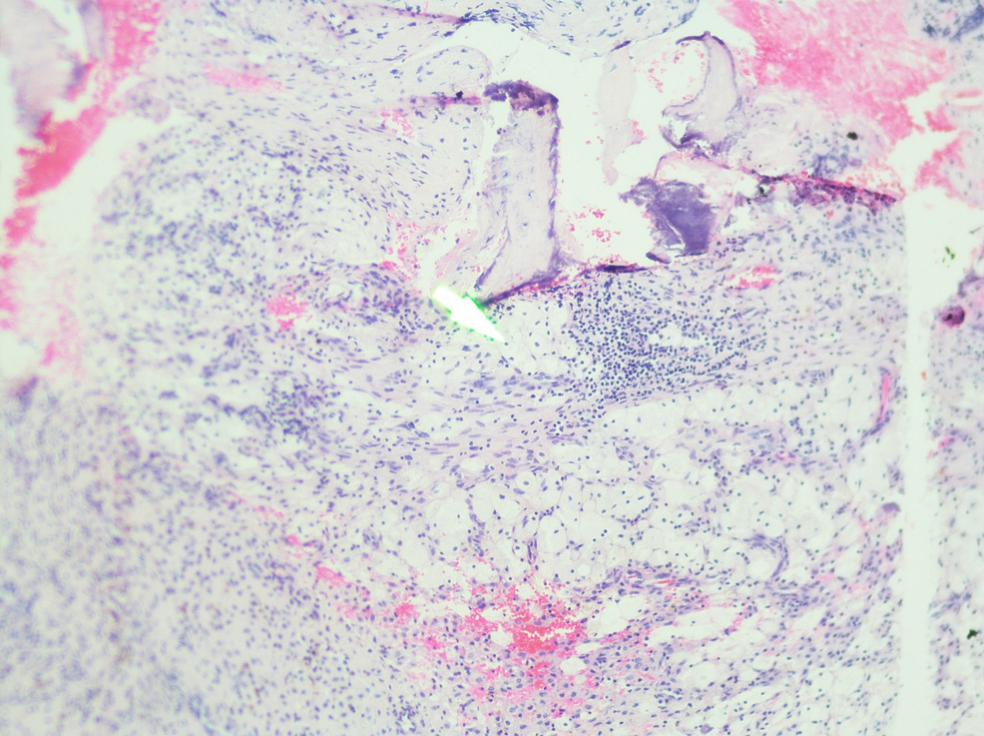
Histiocyte collection with foamy cells, negative for CD1a (white arrow)

**Figure 4:**
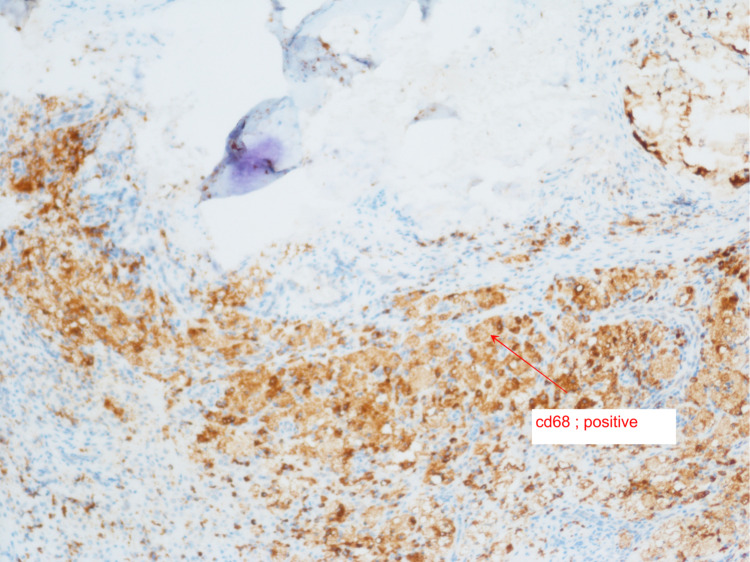
Histiocytes positive for CD68 (red arrow)

**Figure 5:**
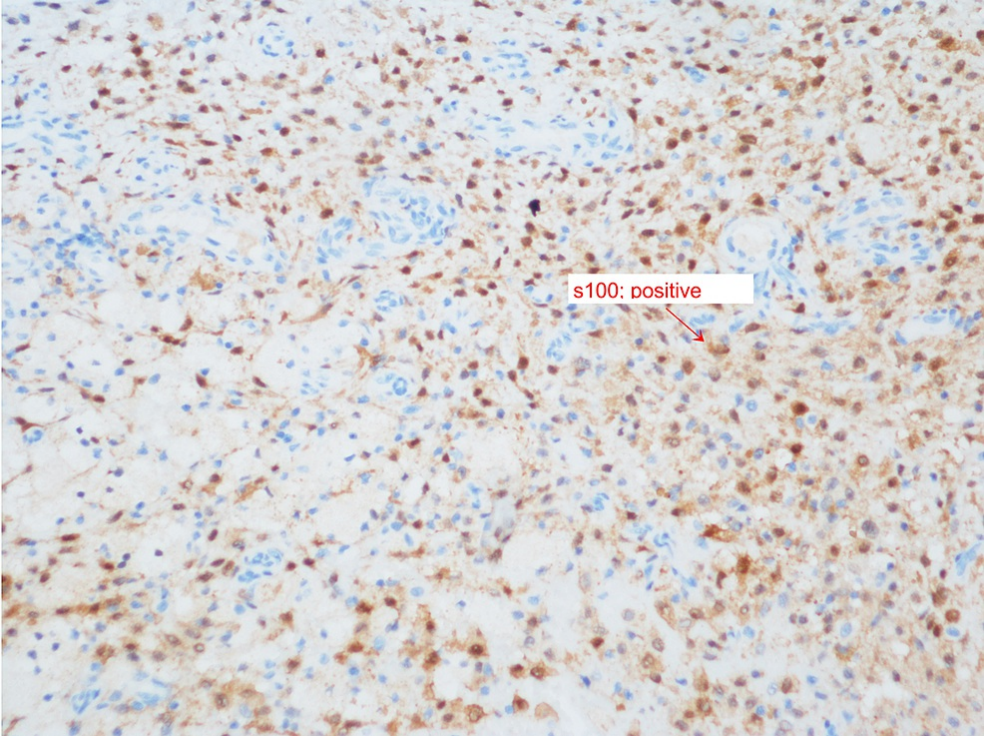
Histiocytes positive for S-100 (red arrow)

The patient was taken to the OR for left mastoid biopsy because the first biopsy was not going with the findings and disease progression; histopathology result showed fibrous tissue with chronic inflammation, calcification, and foamy macrophages. With these findings, we have reviewed the literature for skull base osteomyelitis, cranial nerve palsy, diabetes insipidus, meningitis, and high tibial uptake in scintigraphy scans (Figure 2). Furthermore, we narrowed the possible differential diagnoses to include ECD, Rosai-Dorfman disease, and other differential diagnoses. We targeted the biopsy finding to those with immunochemistry staining (CD68 and S-100) (Figures 3, 4, 5) and correlated it to the clinical and radiological findings; diagnosis of ECD was made, and we managed accordingly.

In the end, this disease was discovered by referring the case to us due to the presence of MOE for further workup, which resulted in discovery of ECD. After a further detailed discussion about the case in the tumor board meeting, the agreed-on decision was to start the patient on interferon-alpha and high-dose cytarabine (HiDAC). The disease showed mild improvement in the beginning but later patient deteriorated and died after six months due to disease aggressiveness with multiple organs involvement.

## Discussion

ECD is a sporadic disease with non-pathognomonic symptomatology and no specific lab findings. Typically, it affects adults between the fifth and seventh decades with a slight predominance in males. The diagnosis is challenging since the disease can present with a wide range of clinical manifestations, which can be seen almost in any body system. The skeletal system is involved in nearly 96% of cases, followed by the CNS in 50% [3]. Genetic and molecular testing for *BRAF* V600E and MAPK pathway mutations helped better understand the disease and revolutionized targeted therapy trials. Biopsies and blood samples for these mutations remain the gold standard for diagnosing ECD, and it is the only common finding to connect all cases of ECD together [3,9]. 

Typically, MOE is seen in the elderly and immunocompromised in around 90% cases [7]. *Pseudomonas aeruginosa* is almost always the causative organism, as described by Grandis et al. [7], unlike our young patient with no known medical illnesses. The patient was not responding well to treatment. He was on IV antibiotics, underwent left mastoidectomy, received targeted immunotherapy like peginterferon, and had four rounds of chemotherapy, and yet, the patient died after six months from the date of diagnosis and targeted treatment which indicates the aggressiveness of the disease.

MRI for brain and sinuses was done and showed bilateral petrous and mastoid destructive changes, with more involvement of the right side. Also, it showed bilateral mastoiditis, bilateral enhancement along membranous labyrinthine. Persistently enhancing and thickened leptomeninges and pachymeninges were noted over the right frontal convexity, with the enhancement and thickening of the tentorial leaflets, thickened pituitary stalk with enhancing and enlarged adenohypophysis, with loss of the spontaneous T1 hyperintensity of the neurohypophysis. There was a bilateral intraocular protrusion of the optic nerve, with no intraocular mass or extraocular muscles abnormality. The patient developed diabetes insipidus and presented later with signs of increased intracranial pressure (ICP), which was supported by MRI findings that showed thickened pituitary stalk, and it was consistent with the systemic review by Cives et al. [10].

It is difficult to differentiate between lateral skull base ECD and Langerhans histiocytosis by imaging since both can show similar findings. Bilateral T2 hypointensity of intraconal orbital lesions and symmetrical cortical osteosclerosis of metaphysis and diaphysis of long bones are characteristic features for ECD. In such cases, differentiation by histologic samples is the mainstay for confirming the diagnosis [6]. In our case, the patient had diabetes insipidus, bilateral hearing loss, severe left sensorineural hearing loss (SNHL), and moderate right hearing loss, and recurrent meningitis. Also, he had a previous history of mastoiditis, facial palsy of the left side, and increased ICP with evidence of bilateral papilledema, visual acuity (VA) of right eye 20/25 and left eye 20/40, and right abducent nerve palsy.

## Conclusions

This is the first reported case of MOE, a typical affection of the elderly, in a 20-year-old patient with ECD. ECD is an infrequent, non-Langerhans histiocytosis disorder. It is a multisystem disease with a poorly understood etiology. We discovered this rare disease when the patient was referred to our tertiary hospital as a case of MOE for further workup. This case highlights the importance of suspecting of MOE even in young patients, especially if risk factors for the disease are present. Early diagnosis can avoid or minimize life-threatening complications.

The clinical features and radiological findings are typical of MOE but even in such a case, especially if the patient is young and without history, it should prompt the treating clinician to investigate further the underlying cause.

## References

[1] Franco-Palacios D, McDonald A, Aguillard RN, Berry A (2017). An unusual case of interstitial lung disease in a patient with cardiopulmonary syndrome as the initial presentation of Erdheim-Chester disease. BMJ Case Rep.

[2] Diamond EL, Dagna L, Hyman DM (2014). Consensus guidelines for the diagnosis and clinical management of Erdheim-Chester disease. Blood.

[3] Mazor R, Manevich-Mazor M, Shoenfeld Y (2013). Erdheim-Chester disease: a comprehensive review of the literature. Orphanet J Rare Dis.

[4] Loureiro B, Altemani A, Reis F (2018). Erdheim-Chester disease with isolated neurological involvement. Radiol Bras.

[5] Goyal G, Heaney ML, Collin M (2020). Erdheim-Chester disease: consensus recommendations for evaluation, diagnosis, and treatment in the molecular era. Blood.

[6] Marinelli JP, Peters PA, Vaglio A, Van Gompel JJ, Lane JI, Carlson ML (2019). Skull base manifestations of Erdheim-Chester disease: a case series and systematic review. Neurosurgery.

[7] Grandis J, Branstetter B, Yu V (2004). The changing face of malignant (necrotising) external otitis: clinical, radiological, and anatomic correlations. Lancet Infect Dis.

[8] Al-Aaraj MS, Kelley C (2020). Malignant Otitis Externa. https://www.ncbi.nlm.nih.gov/books/NBK556138/.

[9] Papo M, Emile JF, Maciel TT (2019). Erdheim-Chester disease: a concise review. Curr Rheumatol Rep.

[10] Cives M, Simone V, Rizzo FM (2015). Erdheim-Chester disease: a systematic review. Crit Rev Oncol Hematol.

